# Suppression of DNA coffee-ring by compacting agents via adsorption at water/substrate and water/air interfaces

**DOI:** 10.1038/s41598-026-52905-w

**Published:** 2026-05-21

**Authors:** Damien Baigl, Mathieu Morel, Sergii Rudiuk

**Affiliations:** CPCV, Department of Chemistry, École Normale Supérieure, PSL University, Sorbonne Université, CNRS, Paris, 75005 France

**Keywords:** Coffee-ring effect, DNA compaction, Polyelectrolytes, Drop evaporation, Interfacial adsorption, Surface patterning, Engineering, Materials science, Nanoscience and technology

## Abstract

The drying of sessile drops of DNA solutions commonly leads to inhomogeneous deposition caused by the so-called coffee-ring effect, which hampers uniform surface immobilization in biosensing and microarray applications. Here, we investigate how DNA-compacting agents influence DNA patterns formed on negatively and positively charged solid substrates upon drop drying. Using poly-L-lysine and spermine as model compacting agents, we demonstrate that the suppression of the coffee-ring effect can arise from two distinct phenomena depending on the compacting agent concentration regime. At low concentrations, compacting agents mainly induce electrostatic adsorption of DNA at the water/substrate interface. Conversely, at higher concentrations, DNA compacts and aggregates, leading to trapping of aggregates at the water/air interface during evaporation and preventing their transport to, and accumulation at, the drop contact line. These findings constitute a new way to suppress the coffee-ring effect through DNA neutralization and compaction, and establish adsorption at interfaces as a general strategy for achieving homogeneous DNA coatings.

**Supplementary Information:**

The online version contains supplementary material available at 10.1038/s41598-026-52905-w.

## Introduction

The coffee-ring effect (CRE) is a ubiquitous phenomenon characterized by the accumulation of non-volatile solutes at the triple contact line of a pinned liquid drop^1–3^. It originates from spatially heterogeneous evaporation of the liquid along the drop radius. Because evaporation is stronger at the contact line than in the center of the drop, compensatory capillary flows of liquid toward the drop edge transport all dissolved or dispersed materials to the contact line, leading to a heterogeneous, ring-shaped deposit after drop drying. The CRE therefore represents a major obstacle to achieving homogeneous deposition of non-volatile compounds from the liquid phase onto solid substrates upon drying^4^. Drying of DNA or protein solutions similarly results in inhomogeneous deposition, which is particularly problematic for the preparation of DNA and protein microarrays. Numerous strategies have been developed to suppress the CRE in general^5^, including preventing pinning or inducing depinning of the contact line^6^, increasing solution viscosity^7,8^, tailoring the shape of colloids^9^, electrowetting^10^, acoustic redispersion during drying^11^, and the generation of competing Marangoni flows^12^. For pinned drops containing spherical colloids, the CRE can also be suppressed without external energy input, simply by preventing the solute from following compensatory flows. This can be achieved by promoting interparticle binding interactions leading to the formation of gel-like structures in the bulk^13^, or by irreversible adsorption of particles at the water/substrate and/or water/air interfaces^14,15^. Notably, efficient suppression of the CRE for microparticles has been achieved by neutralizing their surface charge with surfactants^15,16^, polymers^17^, and even proteins^18^, which promotes their adsorption at the water/air interface during drying.

Here, we investigate whether similar mechanisms can be applied to polyelectrolytes of biological and biotechnological interest, such as DNA. In the dilute regime, long semi-flexible DNA molecules bearing a high negative charge density undergo strong compaction upon neutralization of a critical fraction (89–90%) of their charges^19,20^. Various positively charged agents such as trivalent metal ions and metal complexes^21,22^, organic polyamines^22–24^, polymers^25^, and surfactants^26,27^ are able to neutralize DNA and induce a transition from unfolded coils to compact DNA globules. We thus investigated how neutralization of DNA, changes in its effective size due to compaction, and aggregation of the neutralized DNA influence its deposition patterns formed during drop drying. To this end, we used spermine and poly-L-lysine, two widely used DNA compacting agents, and systematically analyzed their effects on drying patterns of DNA containing drops on different glass substrates.

## Materials and methods

### Materials

λ-phage DNA (48 502 bp), spermine tetrahydrochloride (SPM), poly-L-lysine hydrobromide (4–15 kDa) (PLL), poly(diallyldimethylammonium chloride) 200,000–350,000 Da (PDADMAC), PCR primers, and PBS buffer were from Merck (Sigma-Aldrich). Glass substrates (hydrophobic 24 × 50 mm coverslips made of colorless borosilicate glass (item n°VD12450Y1A.01) and hydrophilic 76 × 26 mm microscope slides made from a special improved soda lime float glass (item n° VA112001FKB.01)) were purchased from Knittel Glass and used without any further treatment. Deionized Milli-Q water (Millipore, 18 MΩ·cm) was used for all experiments.

### Preparation of PDADMAC-treated substrate

Hydrophilic 76 × 26 mm microscope slides were plasma treated for 3 min at 305–340 mTorr air pressure prior to immersing them into a 1 wt% PDADMAC solution in 100 mM NaCl for 2.5 min. The glass slides were then extensively rinsed with Milli-Q water and dried under compressed air flow.

### Preparation of λ-phage DNA

The purchased λDNA solution (556 µg/mL) was desalted three times using G-25 microspin columns. The concentration of the resulting DNA sample was measured with a BioPhotometer Plus spectrophotometer (Eppendorf) in a 10 mm cuvette after 100-fold dilution in 1× PBS buffer. The concentration of the stock desalted λDNA solution was determined to be 67.6 µg/mL (203 µM in phosphate charge).

### λ-phage DNA compaction

λDNA molecules (25 µg/mL) stained with YOYO (0.13 µM) after addition of the required amounts of the compacting agents (PLL or SPM) were observed in 10 µLdrops deposited on a clean glass cover slide. Under these conditions, unfolded and compact DNA molecules are easily distinguishable by both their size and diffusion, enabling direct quantification of the compaction^28–30^. For each condition, a minimum of 200 individual DNA molecules were characterized to determine the fraction of DNA molecules in the compact state.

### Typical drop drying experiment

Desalted DNA, YOYO-1 iodide, and, when necessary, SPM or PLL solutions in deionized water were mixed in this order to obtain a 4 µL solution with final concentrations of [DNA] = 25 µg/mL (76 µM in phosphate charge) and [YOYO] = 0.13 µM. After 30 min of equilibration, 0.8 µL drops of each sample were deposited on the glass surface and allowed to dry at room temperature, protected from the external airflows.

### Fluorescence microscopy

Fluorescence microscopy of the resulting dried patterns was performed using an AxioObserver D1 inverted microscope (Zeiss), equipped with 2.5× and 4× objective lenses. Images were acquired with a highly sensitive EMCCD camera (Photonmax 512B, Princeton Scientific) and MetaVue image acquisition software (Molecular Devices). For bulk observation of individual DNA molecules and DNA compaction, a 100× oil immersion objective lens was used.

### Contact angle measurements

For measurement of static contact angles (*θ*_*static*_), a 0.8 µL water drop was deposited on the corresponding substrate, and side-view images were captured using a CCD camera (asA1600-20uc, Basler) equipped with a 12x zoom lens (MVL12 × 3Z, Thorlabs) and Pylon Viewer software. For measurement of advancing *(θ*_*adv*_) and receding (*θ*_*rec*_) contact angles, a 10 µL Eppendorf pipette tip was positioned in proximity to the substrate, and water was slowly dispensed and withdrawn, respectively, while recording side-view images of the drop. Contact angles were then measured from selected snapshots using ImageJ software.

## Results and discussion

The objective of this study is to establish how DNA compaction could affect its deposition from drying drops. To establish a clear correlation between these two phenomena, we had to define conditions compatible with individual DNA molecule observation in bulk, allowing precise monitoring of compaction, and sufficient material deposition to characterize the deposit properties and the occurrence of the coffee-ring effect (CRE). We chose λ-phage DNA (λDNA), as it is sufficiently long (48 502 base pairs (bp)) to enable optical detection of individual molecules^31,32^, but not too large, as using giant DNA molecules (e.g., T4 DNA) imposes very low concentrations to remain in a dilute regime where individual chains can be distinguished^28–30^. Then, we looked for a concentration range where λDNA is diluted enough to enable single molecule observation, but present in sufficient quantity for the deposition pattern to be detected. Knowing that individual λDNA can be detected at concentrations as low as 1 µg/mL, we varied its concentration from 1 to 50 µg/mL and examined whether the deposition patterns could be analyzed by optical microscopy (Supplementary Fig. S1). By transmission optical microscopy, we could only detect the resulting dried patterns of DNA for the highest used concentration of the nucleic acid (50 µg/mL). Observation of DNA labeled with the fluorescent dye YOYO-1 iodide (YOYO) strongly improved both sensitivity and contrast of DNA detection, allowing us to characterize the patterns from 10 to 50 µg/mL, with particularly fine features observable from 25 to 50 µg/mL. From these observations, and to minimize the amount of DNA for individual molecule observation in bulk, we fixed the DNA concentration at 25 µg/mL, and the YOYO dye concentration at 0.13 µM.

**Fig. 1:**
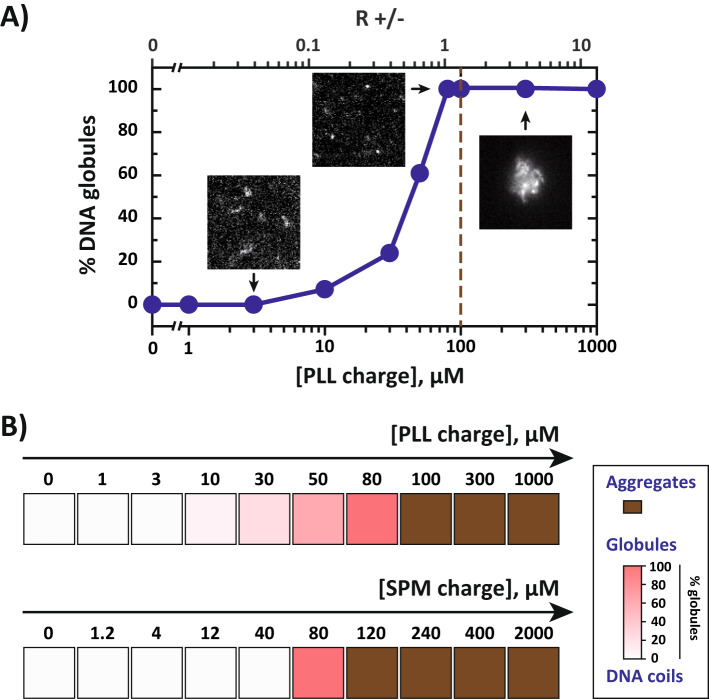
Effect of compacting agents on the morphology of λDNA molecules. (**A**) Typical compaction curve of DNA displaying the percentage of individual DNA molecules in a globule state among the total number, as detected by fluorescence microscopy, as a function of poly-L-lysine (PLL) concentration (in charge). Insets show representative images (31.3 × 31.3 μm) for different states of DNA: unfolded (left), compact (middle) and aggregated (right). The brown dotted line shows the concentration at which individual globules start to aggregate. (**B**) Color code diagrams demonstrating the evolution of DNA in the presence of increasing concentrations of PLL (top) and the tetraamine spermine (SPM, bottom). White color represents 100% unfolded DNA. Levels of red show the percentage (in number) of DNA folded into globules among the total number of detected individual DNA molecules. Brown color corresponds to the formation of aggregated DNA. [λDNA] = 25 µg/mL (76 µM in phosphates); [YOYO] = 0.13 µM in MQ water; room temperature.

Before performing systematic studies of the DNA CRE in the presence of compacting agents, we investigated the DNA compaction at the chosen DNA concentration. We considered two representative compacting agents, which have been widely studied in the literature, each with specific characteristics^20^. The first one was poly-L-lysine hydrobromide (noted PLL), a charged polycation, representative of the interpolyelectrolyte complexation mechanism, and known for its ability to compact DNA at nearly a neutral charge ratio, even in the dilute regime. The second one was the naturally occurring tetramine spermine tetrahychloride (noted SPM), representative of the compaction behavior by small multivalent cations through counter-ion condensation mechanisms. YOYO-labeled DNA was mixed with increasing amounts of each of the compacting agents, and the resulting samples were observed by fluorescence microscopy. Under these conditions, the states of individual DNA are clearly distinguishable: DNA molecules in the compact state appear as bright fast-diffusing spots, whereas DNA molecules in the coil state have a much larger apparent long-axis length, a much lower translational diffusion coefficient and exhibit characteristic intrachain thermal fluctuations^29^. By counting the DNA molecules observed in the compact and unfolded states at each concentration, we established compaction curves, representing the percentage of compact DNA among all detected molecules, as a function of the concentration of the compacting agent. Figure 1A shows a typical DNA compaction curve obtained by adding PLL to the solution of DNA. At [PLL] ≤ 3 µM, the compacting agent was not affecting the DNA conformation and individual λDNA molecules were observed in their native unfolded coil state (Supplementary Video S1). Upon further increase of [PLL], a coexistence region was found where DNA coils coexisted with a progressively increasing amount of compact DNA globules. Finally, the full compaction of the DNA was reached at [PLL] = 80 µM. Interestingly, a slight further increase of the PLL ([PLL] ≥ 100 µM) led to strong aggregation of the DNA globules, leading to the presence of only large, slowly diffusing DNA aggregates in the solution (Supplementary Video S1). Aggregation of compact DNA globules is a well-known phenomenon, especially when working at high DNA concentrations, and is mainly attributed to the loss of stability of DNA upon its neutralization^33,34^. The same behavior was observed for DNA compaction with tetracharged SPM (Supplementary Video S2). Right after the full compaction of DNA at [SPM] = 20 µM, the globules started to aggregate for [SPM] ≥ 30 µM (Fig. 1B). For the used concentration of DNA phosphate charges (76 µM), full compaction was observed for 80 µM of PLL monomers and 20 µM of tetraamine SPM. Interestingly, this corresponded to the charge ratio R_+/−_ = [compacting agent charge]/[DNA phosphates] ≈ 1 in both cases, confirming the predominance of the electrostatic neutralization in driving DNA compaction at high DNA concentrations^26^.

**Fig. 2:**
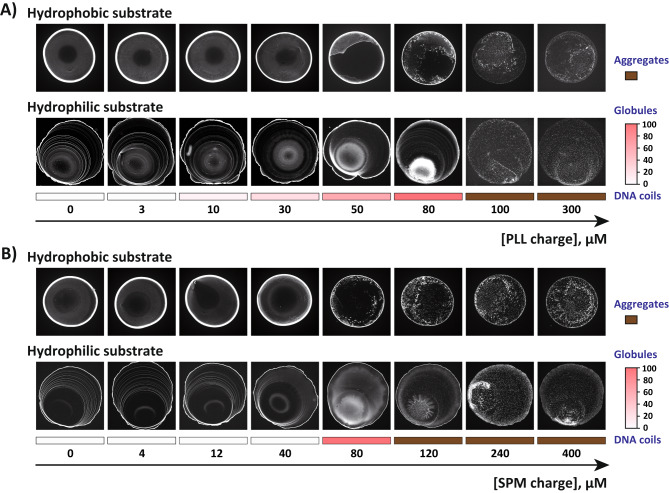
Effect of compacting agents on deposit morphology of drying sessile DNA drops. Typical patterns obtained after drying of 0.8 µL MQ water drops containing 25 µg/mL λDNA (76 µM in phosphates) in the presence of increasing concentrations of PLL (**A**) and SPM (**B**) on hydrophobic (top) and hydrophilic (bottom) glass slides. Color bars indicate the corresponding state of DNA at each concentration of the compacting agents. The images are 3.54 × 3.54 mm and 2.24 × 2.24 mm on hydrophilic and hydrophobic substrates, respectively.

We next investigated the drying patterns of DNA in the presence of increasing concentrations of compacting agents on two kinds of glass substrates showing different wetting behaviors for pure water: a soda lime float glass substrate with a static contact angle of 38°, referred to as “hydrophilic” and a borosilicate glass with a significantly larger static contact angle of 75°, referred to as “hydrophobic” (Supplementary Fig. S2 and Table S1). To establish the direct parallel between DNA compaction and deposition pattern behaviors, we rigorously kept the same conditions used for Fig. 1A (DNA and dye concentrations) and, for each sample, we deposited a 0.8 µL drop on each substrate and let it dry protected from air flows. Figure 2A shows the obtained patterns with PLL as the compacting agent. In absence of PLL, a typical single coffee-ring was observed on the hydrophobic substrate, due to the pinning of the drops, whereas a multiring pattern formed on the hydrophilic substrate. The latter behavior has been reported for the drying of concentrated DNA solutions^35,36^ and arises from oscillatory stick-slip motion of the receding contact line due to the competition between friction and surface tension at the contact line^37^. Interestingly, in both cases, addition of PLL strongly affected the drying patterns of DNA. A sharp transition was observed on the hydrophobic substrate where the single coffee ring persisted as long as [PLL] < 100 µM, but was suppressed for [PLL] ≥ 100 µM (Fig. 2A, top). Notably, this concentration corresponded to the onset of the aggregation of compact DNA globules, indicating a correlation between the evolution of DNA states and that of the deposition pattern. The multiring pattern on the hydrophilic substrate followed a more gradual evolution with increasing PLL concentration, and was correlating particularly well with the evolution of the compaction state (Fig. 2A, bottom). In this case, even at PLL concentrations corresponding to a low fraction of compact DNA, the multiring structure was altered, with progressive widening of the rings and homogenization of the inner region of the deposit following the increase of the fraction of compact DNA (Fig. 2A, bottom). The onset of aggregation of the compact DNA matched the complete suppression of the outer ring, in striking similarity with the behavior observed on the hydrophobic surface. All these results not only show how the addition of a cationic polyelectrolyte affects the deposition pattern of a drying DNA drop up to suppression of the CRE, but also reveal a strong correlation with the neutralization/compaction behavior of DNA.

To investigate the generalizability of the phenomenon observed with PLL, we performed the same study but using spermine (SPM) as the DNA compacting agent (Fig. 2B). Interestingly, although SPM is chemically and structurally very different from PLL, and involves a different mechanism for DNA compaction^20^, some similarities were observed in the evolution of the deposition pattern. Like for PLL, the pattern evolution was sharp on the hydrophobic substrate (Fig. 2B, top) and more progressive on the hydrophilic one (Fig. 2B, bottom), with a suppression of the coffee-ring effect corresponding to the appearance of globules. This confirms the correlation between the evolution of DNA upon neutralization by compacting agent and that of the deposition pattern, independent of the nature of the compacting agent. However, a few notable differences were observed. The homogenization on the hydrophilic substrate was also observed for lower SPM concentration ([SPM] = 40 µM in charge), for which DNA remained largely unfolded, indicating that the DNA compaction state alone cannot account for the deposition pattern properties. To further investigate the underlying mechanism, we compared drying patterns of DNA molecules of another length (206 bp) at various SPM concentrations (Supplementary Fig. S3). No differences were observed between short synthetic 206 bp DNA and the large λDNA (48.5 kbp), confirming an important role of electrostatic interactions in the observed effects.

**Fig. 3:**
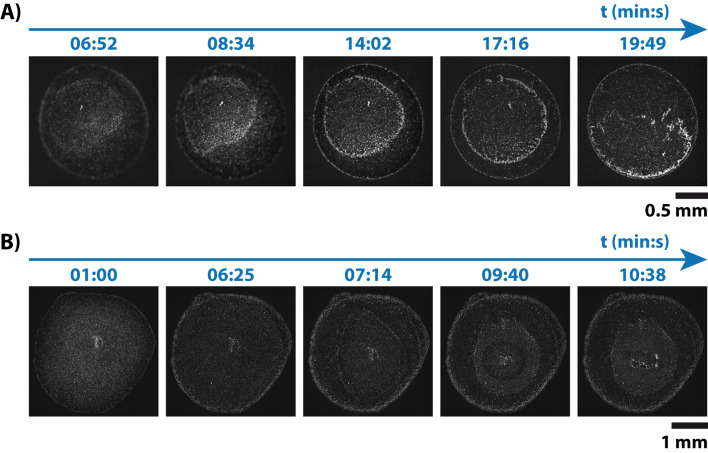
Observation of adsorption of DNA-PLL aggregates at the water/air interface during drop drying. Fluorescence microscopy images of a 0.8 µL sessile drops drying on the hydrophobic (**A**) and hydrophilic (**B**) substrates as a function of time (t = 0 corresponds to the drop deposition on the substrate). In (**A**) the focus was manually adjusted on the apex of the drop, whereas in (**B**) it was kept constant. The last images correspond to the fully dried patterns. [λDNA] = 25 µg/mL, [YOYO] = 0.13 µM, and [PLL] = 100 µM in MQ water.

CRE is mainly controlled by hydrodynamic effects and flow patterns^38^, interactions^14^, or a combination of both. In our case, there is no change of physical parameters that could modify the flow patterns. Therefore, the observed homogenization of dried DNA patterns can be attributed to interactions. As DNA is a negatively charged polyelectrolyte, multivalent cationic agents facilitate its adsorption on negatively charged glass substrates^39–41^. Such compacting agent-mediated adsorption at low concentrations thus represents a powerful means for the homogenization of DNA drying patterns. Adsorption at the water/substrate interface alone cannot, however, account for the complete suppression of the coffee-ring effect observed at high concentrations of the compacting agents, i.e., for aggregated DNA. Even though precipitation of the aggregates can take place, owing to their low diffusion coefficients, large DNA aggregates are expected to have lower probability of diffusing toward the substrate and should rather be transported toward the contact line by compensatory capillary flows. We thus directly monitored the water/air interface during evaporation of the drops containing aggregated DNA ([PLL] = 100 µM) on both substrates using fluorescence microscopy (Fig. 3).

Figure 3A and Supplementary Video S3 show fluorescence microscopy observation of the apex of a drying DNA drop in presence of PLL on the hydrophobic substrate. After several minutes of evaporation, a large number of DNA aggregates were progressively adsorbed at the water/air interface and were deposited on the substrate at the final stage of evaporation. The trapping of these aggregates at the interface hindered their transport by the evaporation-driven flow toward the contact line, resulting in their deposition within the inner region of the final dried pattern. A similar behavior of DNA aggregates was observed during drying on the hydrophilic substrate, except that the adsorbed DNA was deposited more gradually throughout the interior of the pattern as a result of progressive unpinning of the contact line before complete evaporation (Fig. 3B, Supplementary Video S4). In both cases, adsorption of DNA aggregates at the water/air interface thus resulted in homogenization of the drying DNA material. All these results are consistent with our previous findings that coffee-ring effect of nanoparticles can be suppressed by their trapping by the descending water/air interface upon drying due to the neutralization and hydrophobization of their surface by surfactants or proteins^14,16,18^. Notably, this study provides the first evidence that such interfacial trapping can also occur at a more molecular level, as exemplified here with neutralized and aggregated DNA molecules.

**Fig. 4:**
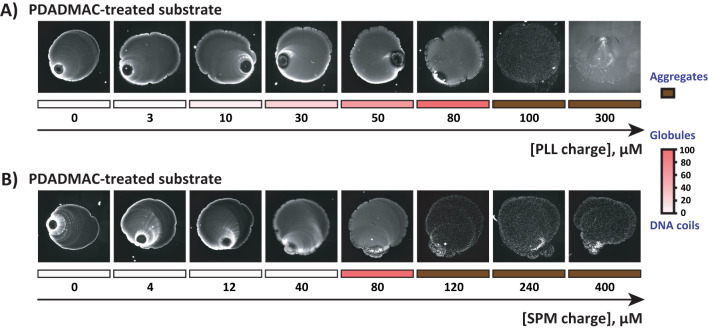
Effect of compacting agents on deposit morphology of drying sessile DNA drops on positively charged substrate. Typical patterns obtained after drying of 0.8 µL MQ water drops containing 25 µg/mL λDNA (76 µM in phosphates) in the presence of increasing concentrations of PLL (**A**) and SPM (**B**) on PDADMAC-treated positively charged glass slides. Color barsindicate the corresponding state of DNA at each concentration of the compacting agents. The images are 4.97 × 4.97 mm.

The previous results showed that two adsorption phenomena must be taken into account to explain the deposition behavior: (i) at low compacting agent concentrations, the adsorption of DNA at the water/substrate interface before full DNA compaction; and (ii) at high compacting agent concentrations, the adsorption of aggregated DNA at the water/air interface. In the latter case, however, we could not exclude a possible role of the adsorption of aggregated DNA on the solid substrate. To disentangle these two possible mechanisms, we investigated drying pattern formation on positively charged, hydrophilic glass substrates functionalized with poly(diallyldimethylammonium chloride) (PDADMAC). Because this polymer contains fully quaternized amines, electrostatic adsorption of compacted DNA at the substrate is expected to be limited, and it was therefore interesting to assess whether the CRE could be suppressed as well. Interestingly, a behavior similar to that on bare glass was observed for DNA compaction both with PLL (Fig. 4A) and SPM (Fig. 4B). Although increased hydrophilicity of the substrate led to larger drop diameters, characteristic multiring patterns were still present. At low concentrations of the compacting agents, adsorption of unfolded DNA on the positively charged substrate partially homogenized the deposits, resulting in a DNA concentration gradient, due to differences in adsorption times arising from the stick-slip motion of the receding contact line, while an outer coffee-ring still remained. As compaction progressed, both features disappeared, yielding rather uniform DNA deposits. After DNA aggregation, homogeneous distribution of the aggregates, comparable to those found on negatively charged glass was observed. Together, these results indicate that adsorption at both the water/air and water/substrate interfaces suppresses the CRE, with water/air interfacial adsorption playing a dominant role in homogenizing compacted and aggregated DNA upon drying. They also provide a practical way for obtaining homogeneous DNA deposition upon drying through the combined use of compacting agents and hydrophilic positively charged solid substrates.

## Conclusions

In summary, we investigated the influence of DNA compacting agents on patterns formed upon drying sessile drops of DNA solutions and demonstrated that two distinct effects could be clearly distinguished in this system. At low concentrations of compacting agents, adsorption of DNA on negatively-charged substrates occurs even before compaction due to the multivalent cationic nature of the compacting agents. Conversely, compact DNA and DNA aggregates formed at high compacting agent concentrations are trapped at the water/air interface due to the affinity of neutralized DNA for hydrophobic interfaces. Both phenomena lead to a decrease in DNA deposition at the contact line, leading to more homogeneous deposition and ultimately completely suppressing the coffee-ring effect at high compacting agent concentrations. The reported trapping of compacted DNA at the water/air interface during drop evaporation extends known mechanisms of coffee-ring suppression of DNA. Our results highlight the crucial role of interfacial adsorption in directing DNA deposition and provide a simple strategy to obtain uniform coatings of polyelectrolytes of biological interest, with potential to improve biosensing platforms, microarray technologies and other applications requiring homogeneous DNA surface immobilization.

## Supplementary Information

Below is the link to the electronic supplementary material.

Supplementary Material 1

Supplementary Material 2

Supplementary Material 3

Supplementary Material 4

Supplementary Material 5

## Data Availability

The datasets generated and/or analysed during the current study are available from the corresponding authors on reasonable request.
