# Supplementary material for: Suppression of DNA coffee-ring by compacting agents via adsorption at water/substrate and water/air interfaces
Source: Sci Rep. 2026 May 21;16:23255. doi: 10.1038/s41598-026-52905-w (PMC13402349; doi:10.1038/s41598-026-52905-w)
Supplement: Supplementary file 5 — Supplementary Material 5 [file 41598_2026_52905_MOESM5_ESM.pdf]

Supplementary information

Suppression of DNA coffee-ring by compacting agents via adsorption at water/substrate and water/air interfaces

Damien Baigl*, Mathieu Morel and Sergii Rudiuk*

CPCV, UMR8228, Department of Chemistry, PSL University, Sorbonne Université, CNRS, Ecole Normale Supérieure, 75005 Paris, France

Corresponding authors' emails: sergii.rudiuk@ens.psl.eu and damien.baigl@ens.psl.eu

Preparation and sequence of the 206 bp DNA (5’-3’)

Preparation of 206 bp DNA

A 206 bp DNA fragment was prepared by PCR using the pAG001 plasmid as the template (a gift from A. Gautier, ENS), with TTACGGTAAACTGCCCACTTG (forward) and CCACGCCCATTGATGTACTGC (reverse) primers. Reaction mixtures (50 µL) were prepared on ice with the following final composition: 0.2 mM dNTPs, 50 ng DNA template, 0.5 µM primers, and 0.5 µL of 2000 U mL^{-1} Phusion High-Fidelity DNA polymerase in HF buffer (New England BioLabs Inc.). After an initial step at 98 °C for 30 s, 25 PCR cycles (98 °C for 10 s, 66 °C for 20 s, and 72 °C for 10 s) were performed, followed by a final elongation step at 72 °C for 5 min. The length and purity of the amplified DNA were checked by agarose gel electrophoresis. Finally, the amplified 206 bp DNA was purified using a QIAquick PCR Purification Kit (QIAGEN), eluted in deionized water, and its concentration was determined by absorbance using a BioPhotometer Plus spectrophotometer (Eppendorf).

Sequence of 206 bp DNA

TTACGGTAAACTGCCCACTTGGCAGTACATCAAGTGTATCATATGCCAAGTACGC
CCCCTATTGACGTCAATGACGGTAAATGGCCCGCCTGGCATTATGCCCAGTACAT
GACCTTATGGGACTTTCCTACTTGGCAGTACATCTACGTATTAGTCATCGCTATTA
CCATGGTGATGCGGTTTTGGCAGTACATCAATGGGCGTGG

Supplementary Figures

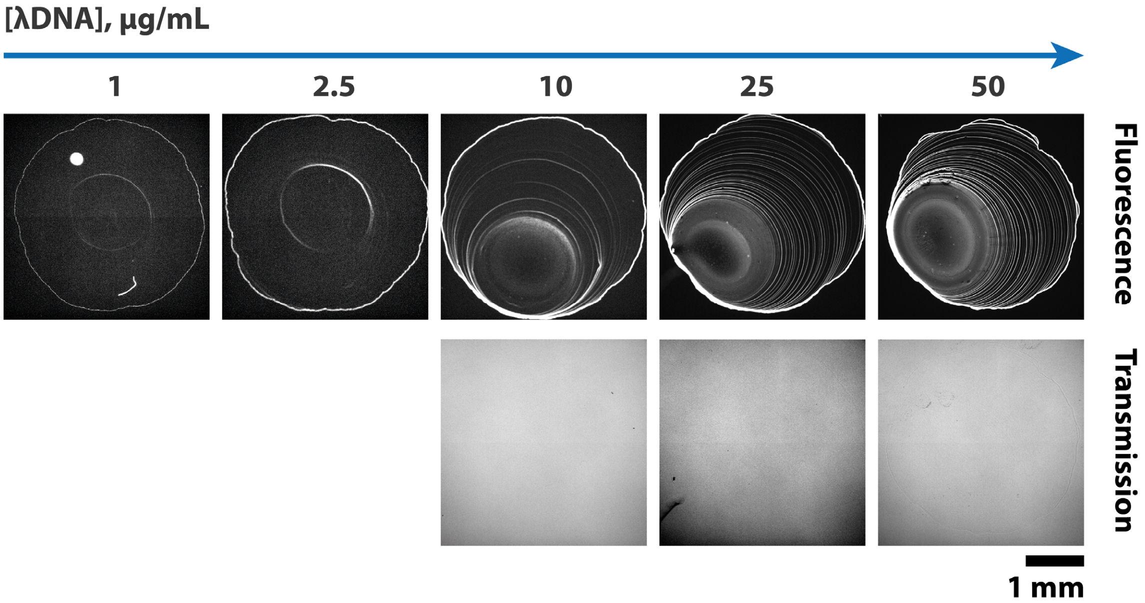

Figure S1. Drying patterns on hydrophilic glass slides for different concentrations of λDNA observed by fluorescence (top) and transmission (bottom) optical microscopy. [λDNA]/[YOYO] = 585; MQ water; room temperature.

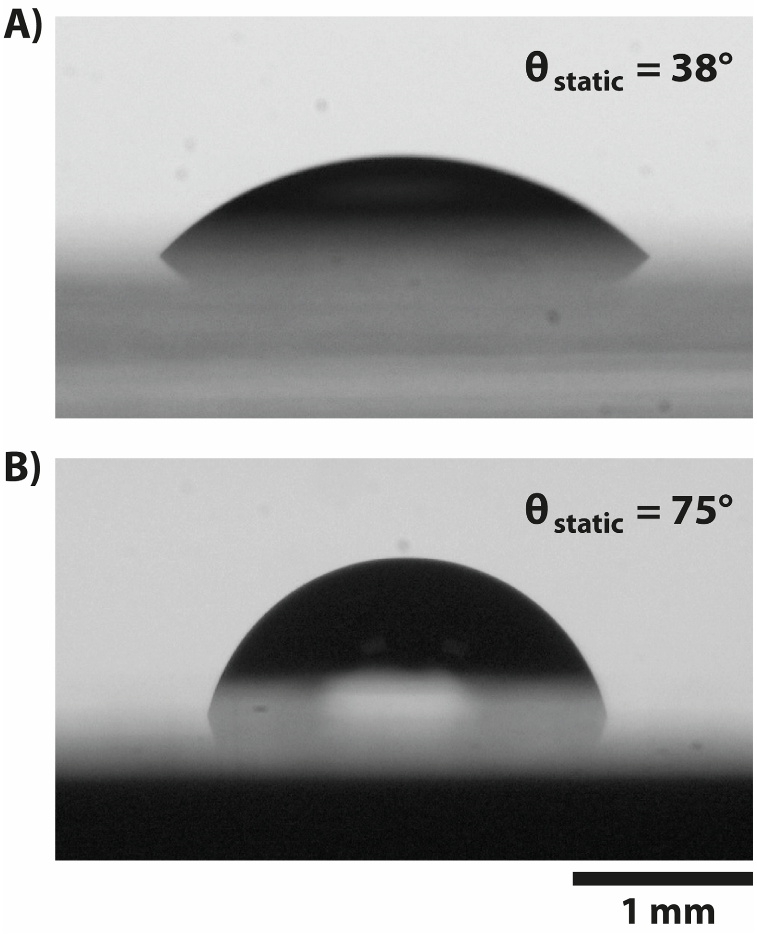

Figure S2. Side view of 0.8 µL water drops deposited on hydrophilic (A) and hydrophobic (B) bare glass slides. ϑ_{static} represents the measured static contact angles.

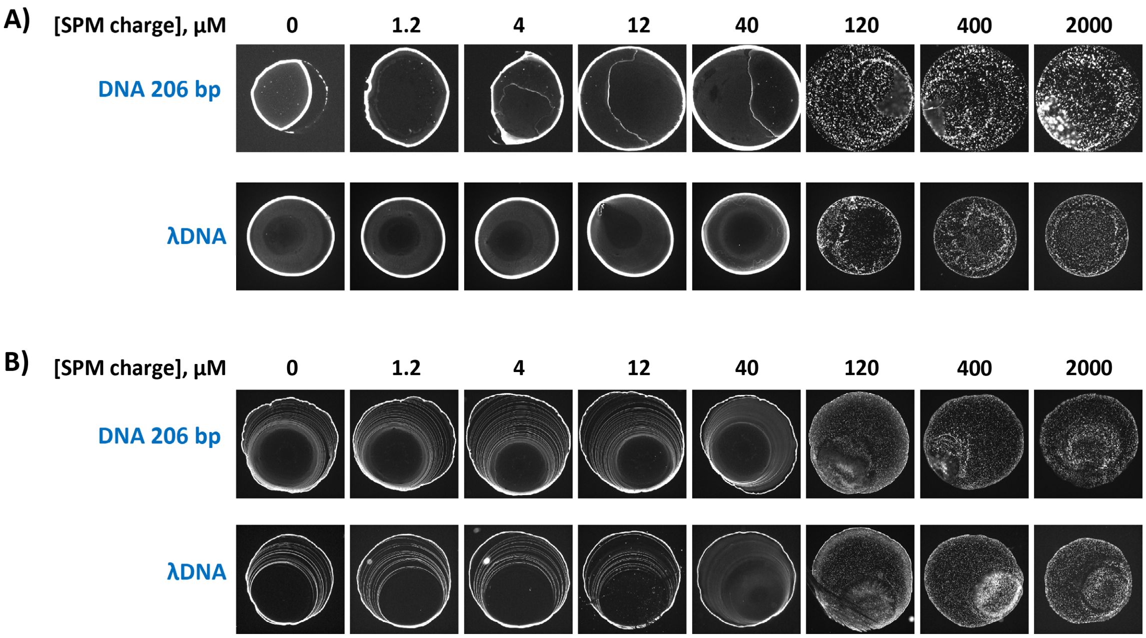

Figure S3. Comparison of drying patterns on hydrophobic (A) and hydrophilic (B) glass slides for short synthetic 206 bp DNA and giant genomic λDNA (48.5 kbp) in the presence of increasing concentrations of SPM.

Supplementary Table

Table S1. Measured values of advancing (ϑ_{adv}), receding (ϑ_{rec}) and static (ϑ_{static}) contact angles for pure water on the used hydrophilic and hydrophobic bare glass slides.

	ϑ_{adv}	ϑ_{rec}	ϑ_{static}
**Hydrophilic glass slide**	38°	13°	38°
**Hydrophobic glass slide**	78°	44°	75°

Video Captions

Video S1. Real time videos of fluorescence microscopy observation of λDNA (48.5 kbp) upon addition of increasing concentrations of poly-L-lysine (PLL) in deionized water. [λDNA] = 25 μg/mL, [YOYO-1 iodide] = 0.13 μM.

Video S2. Real time videos of fluorescence microscopy observation of λDNA (48.5 kbp) upon addition of increasing concentrations of spermine (SPM) in deionized water. [λDNA] = 25 μg/mL, [YOYO-1 iodide] = 0.13 μM.

Video S3. Fluorescence microscopy observation of the drying process of λDNA (25 μg/mL) aggregated with 100 μM in charge of PLL in MQ water on hydrophobic substrate. The video is accelerated, and the time scale is depicted at the top right corner as min:s. [YOYO-1 iodide] = 0.13 μM. Starting from minute 4 the focus was manually positioned at the apex of the drying droplet.

Video S4. Fluorescence microscopy observation of the drying process of λDNA (25 μg/mL) aggregated with 100 μM in charge of PLL in MQ water on hydrophilic substrate. The video is accelerated, and the time scale is depicted at the top right corner as min:s. [YOYO-1 iodide] = 0.13 μM. The focal point is constantly positioned at the surface of the substrate.
